# Six pairs of things to celebrate on International Clinical Trials Day

**DOI:** 10.1186/1745-6215-14-128

**Published:** 2013-05-20

**Authors:** David L Sackett

**Affiliations:** 1 Trout Research & Education Center, Markdale, ON, N0C 1H0, Canada

## Abstract

The Editor has invited me to mark the occasion with a few words “on the development and/or impact of randomised trials over the last decade, or perhaps some thoughts about the future.” I do so here by citing some recent developments that give me cause for both pride about the present state of randomised controlled trials (RCTs) and optimism about their future. Respecting the sample size of James Lind’s trial and the space allotted me by the Editor, they appear in six pairs.

## 

I designed my first randomised controlled trial (RCT) in 1967, the same year that Tom Chalmers suggested that sharing the unblinded interim results of the Coronary Drug Project with all its investigators was a bad idea [1]. PubMed lists 218 RCTs published that entire year, equivalent to just three days output in 2012. In 1967, we didn’t have a lot to celebrate about RCTs beyond their elegant simplicity and the small band of heroes who were applying them to highly controversial questions such as: Might giving antihypertensive drugs to symptomless U.S. veterans with diastolic blood pressures between 115 and 129 mmHg do more good than harm [2]?

Forty-six years and half a million RCTs later, we have so much to celebrate about RCTs that we observe an annual International Trials Day on or about the 20^th^ of May, the day in 1747 that James Lind started giving daily doses of cider, sulphuric acid, acetic acid, sea water, two oranges plus a lemon, or nutmeg to six different pairs of scorbutic sailors with “putrid gums, the spots and lassitude, with weakness in the knees” [3].

The Editor has invited me to mark the occasion with a few words “on the development and/or impact of randomised trials over the last decade, or perhaps some thoughts about the future.” I do so here by citing some recent developments that give me cause for both pride about the present state of RCTs and optimism about their future. Respecting the sample size of James Lind’s trial and the space allotted me by the Editor, they appear in six pairs.

1.1. *Patients are increasingly setting RCT agendas.*

Patient groups are increasingly holding trialists’ feet to the fire and forcing us to honour our obligations to them and their diseases, illnesses, predicaments, and other patient-relevant outcomes, rather than focusing only on our interests and those of the drug and device industries. Its most progressive form is seen in the James Lind Alliance [4], which “brings patients, carers and clinicians together to identify and prioritise the top 10 uncertainties, or ‘unanswered questions’, about the effects of treatments that they agree are most important.”

1.2. *Claims that participants in RCTs are mere sacrificial guinea pigs are being refuted.*

Systematic reviews of studies of similar patients treated inside and outside RCTs are continuing to dispel this myth [5].

2.1. *Low-income country RCTs are increasing in number, local relevance, and international recognition.*

Among growing numbers of examples, a trial of pre- and post-natal education delivered by neighbours in one of the poorest regions of India not only reduced neonatal mortality and post-partum depression, but also was recognised as the “Trial of the Year” by the Society for Clinical Trials [6].

2.2. *Low-income country RCTs are increasingly led by local trialists.*

Ever more of their heroes are not only leaving home to get trained as trialists but also returning home to collaborate with their compatriots, both in carrying out locally-relevant trials and in collaborating in multinational trials of local relevance.

3.1. *Trial questions and protocols are increasingly being disclosed prior to data acquisition and massage.*

Trial registration has become the rule, and the pre-trial publication of protocols is discouraging (or at least exposing to ridicule and approbation) data-dependent disappearances of trial participants and their events.

3.2. *The notion of widely sharing RCT results is gaining momentum.*

The development of policies and procedures that will protect validity and defeat distortion while opening access is beginning to overcome the scepticism and resistance (including my own) to this notion.

4.1. *Ethics committees are under increasing scrutiny for the harm that they do.*

For example, their inconsistent demand for informed consent at some but not other centres in the CRASH-2 trial of tranexamic acid for major trauma was shown to delay treatment by about an hour and result in unnecessary deaths [7]. Similarly, a group of Australian cancer trialists calculated that delays in multicentre ethics approval were responsible for 60 cancer deaths per year [8].

4.2. *Ethics committees are working to reduce the harm that they do.*

For example, 25 of 27 Ontario institutions that conduct multi-centre cancer clinical trials have delegated their ethics review to a single centralised expert oncology research ethics board [9]. It meets every month, and trials it approves can begin to recruit the next day.

5.1. *Frequentist statisticians are continuing to create innovative approaches to the design and analysis of RCTs,* such as multi-arm, multi-stage trials [10].

5.2. *Bayesian statisticians are continuing - with remarkably good humour - their Sisyphean efforts to educate the rest of us about better ways to think about and carry them out*[11]*.*

6.1. *The Cochrane Collaboration continues to flourish.*

Generating the ‘gold standard’ for determining the effects of health care, its more than 28,000 dedicated people from over 100 countries have prepared, updated, and promoted the accessibility of over 5,000 Cochrane Reviews.

6.2. *So are CONSORT*[12]*and PRISMA/QUOROM*[13]*.*

## Competing interest

Dave Sackett has been wined, dined, supported, transported, and paid to speak by countless pharmaceutical firms for over 50 years, beginning with two research fellowships and interest-free loans that allowed him to stay to finish medical school. Dozens of his randomised trials have been supported in part (but never in whole) by pharmaceutical firms, who never received or analysed primary data and never had veto power over any reports, presentations, or publications of the results. He has twice worked as a paid consultant to advise pharmaceutical firms whether their products caused lethal side-effects; on both occasions he told them “yes.” He has testified as an unpaid expert witness for a stroke victim who successfully sued a manufacturer of oral contraceptives, and as a paid expert in preparing individual suits against manufacturers of female hormones and NSAIDs and in class-action suits against manufacturers of prosthetic heart valves and oral contraceptives. He was paid by a pharmaceutical firm to develop “levels of evidence” for determining the causation of adverse drug reactions. His wife inherited and sold stock in a pharmaceutical company. While head of a division of medicine he enforced the banning of drug-detail personnel from clinical teaching units (despite the threat of withdrawal of drug industry funding for resident research projects). He received the Pharmaceutical Manufacturers’ Association of Canada Medal of Honour (and cash) for “Contributions to Medical Science in Canada” for the decade 1984–1994. A recent award (the 2010 Gairdner-Wightman Award) was sponsored by the Canadian Government and 21 research bodies, provinces, and manufacturers.
